# Inhibition of mtor in kidney cancer

**DOI:** 10.3747/co.v16i0.419

**Published:** 2009-05

**Authors:** A. Kapoor

**Keywords:** Temsirolimus, everolimus, sirolimus, mtor inhibitors, renal cell carcinoma, kidney cancer

## Abstract

The mammalian target of rapamycin (mtor) has been shown to be an important target mechanism in the treatment of renal cell carcinoma (rcc). In first-line treatment for patients with disease having poor prognostic features, temsirolimus, an mtor inhibitor approved for treatment of advanced rcc, has demonstrated benefit over interferon alfa in both overall and progression-free survival. Everolimus, a second mtor inhibitor that has showed activity in rcc, led to improved progression-free survival in a comparison with placebo in patients whose rcc progressed after treatment with vascular endothelial growth factor receptor tyrosine kinase inhibitors (sunitinib, sorafenib, or both). There is now compelling clinical evidence for the effectiveness of targeting mtor in the treatment of rcc.

## 1. INTRODUCTION

Over the last few years, treatment for metastatic renal cell carcinoma (mrcc) has undergone a paradigm shift from the mainstay of cytokine therapy (interferon alfa and interleukin-2) to new targeted therapies, including multiple receptor tyrosine kinase (rtk) inhibitors and mammalian target of rapamycin (mtor) inhibitors. The new rtk inhibitors, which include sunitinib (Sutent: Pfizer Canada, Kirkland, QC) and sorafenib (Nexavar: Bayer HealthCare AG, Leverkusen, Germany), interfere with tumour-cell proliferation and angiogenesis. Sunitinib and sorafenib have both shown efficacy in patients with advanced good- to intermediate-prognosis rcc. Sunitinib has demonstrated activity as a first-line therapy for mrcc 1, and sorafenib has demonstrated activity in patients with failure of prior cytokine therapy 2. As compared with interferon alfa, the mtor inhibitor temsirolimus (CCI-799; Torisel: Wyeth, Madison, NJ, U.S.A.) has been shown to prolong overall survival (os) in previously untreated poor-prognosis patients with advanced rcc 3.

## 2. THE mTOR PATHWAY

A substantial body of research, including clinical and preclinical studies, has identified a central role for the mtor signalling pathway in cell growth, proliferation, survival, and angiogenesis. Aberrations of mtor-mediated signalling pathways and mtor upregulation have been discovered in many different tumour types, establishing the mtor pathway and its inhibition as a promising treatment target in human cancers.

Multiple stimuli such as growth factors [for example, epidermal growth factor (egf), platelet-derived growth factor (pdgf), and insulin-like growth factor (igf)], nutrients, oxygen, and stress can lead to activation of the mtor pathway and downstream signalling.

The mtor kinase, the central regulator of the mtor signalling pathway, is a sizeable (289 kDa) polypeptide serine/threonine–specific kinase that is part of the phosphoinositide 3 kinase (pi3k)–related kinase family 4. By regulating general protein biosynthesis, mtor kinase is involved in the control of a wide variety of growth-related cellular functions such as transcription, translation, membrane trafficking, protein degradation, and reorganization of the actin cytoskeleton 4,5.

### 2.1 Upstream Activation of mTOR Signalling

Signalling through the mtor pathway starts with the receipt of cell growth and survival signals relayed to the internal cellular environment by rtks on the plasma membrane. Activation of protein kinase B [Akt (a serine/threonine protein kinase)] by pi3k is the key event in the signalling cascade. It results from an accumulation of phosphatidylinositol (3,4,5)-trisphosphate (pip3) formed by pi3k from phosphatidylinositol 4,5-bisphosphate (pip2), which leads to phosphorylation of Akt.

Once substantially accumulated, the phospholipid pip3 serves as a source for recruiting kinases to the plasma membrane, including the Akt family of kinases and pyruvate dehydrogenase kinase 1 (pdk1). The pdk1 activates Akt, partly through phosphorylation, and Akt subsequently phosphorylates a number of downstream targets, including mtor kinase, whose increasing phosphorylation by Akt further enhances protein synthesis. These Akt targets play key roles in regulating critical cellular functions including proliferation, apoptosis, glucose homeostasis, cell size, nutrient response, and dna damage 6–8.

In normal tissues, the tumour suppressor gene *PTEN* (phosphatase and tensin homologue deleted on chromosome 10) is a negative regulator of the Akt pathway, acting by downregulating pi3k hyperactivity through dephosphorylation of pip3 to pip2. In many cancers, this *PTEN* tumour suppressor function is not active, allowing pi3k to activate Akt. There is substantial evidence that inactivation of *PTEN* may be more important than mutation of *p53* in many adult epithelial tumours^9^. Somatic alteration and mutation of *PTEN* has been shown to be a common event in tumours such as melanoma, glioblastoma, prostate cancer, and endometrial cancer 10.

**FIGURE 1:**
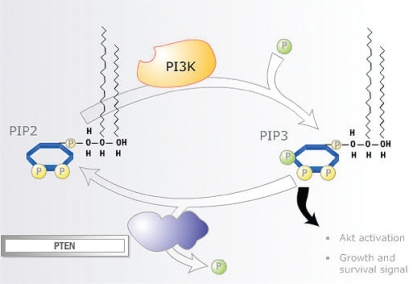
The phosphoinositide 3 kinase (pi3k)/protein kinase B (Akt) signalling pathway 10. pip2 = phosphatidylinositol (4,5)-bisphosphate; pip3 = phosphatidylinositol (3,4,5)-trisphosphate; pten = phosphatase and tensin homolog.

The pten protein encoded by the *PTEN* gene is an enzyme that facilitates dephosphorylation of pip3 to pip2. With growth-factor stimulation (igf, egf, pdgf), pip3 is upregulated. Elevated pi3k has been linked to transformation by oncogenes and to stimulation through the pdgf receptor. The lipid phosphatase activity of pten and its ability to dephosphorylate pip3 and act as a “countermeasure” for pi3k signalling suggests that pten functions as a significant tumour suppressor by directly antagonizing the activity of the pi3k/Akt signalling pathway (Figure 1).

**FIGURE 2:**
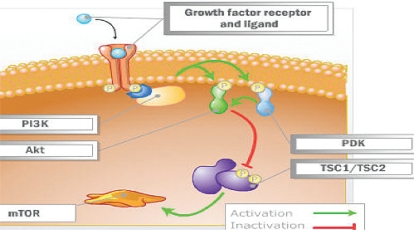
Stimulation of protein kinase B (Akt) by growth factors and mitogens in a pi3k (phosphoinositide 3 kinase)–dependent manner phosphorylates and subsequently destabilizes the tuberous sclerosis (Tsc1/Tsc2) complex. 10 pdk = pyruvate dehydrogenase kinase; mtor = mammalian target of rapamycin.

Significant research has now shown the tumour suppressor genes tuberous sclerosis 1 (*TSC1*) and 2 (*TSC2*) to be key inhibitors of the mtor pathway 5. After Akt is stimulated by growth factors and mitogenic stimuli through the pi3k pathway, the Akt complex phosphorylates and subsequently destabilizes the tsc complex (Figure 2). This destabilization of the tumor suppressors tsc1 and tsc2 subsequently activates mtor via a small protein called Rheb (*ras* homolog enriched in brain).

### 2.2 Dual Pathway Activity: Downstream mTOR Signalling Targets

**FIGURE 3:**
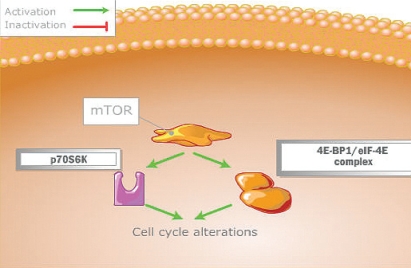
Control of the cell replication process by mammalian target of rapamycin (mtor) through two downstream pathways 10. 4EBP1/eIF-4E = eukaryotic translation initiation factor 4E binding protein 1 / eukaryotic translation factor; p70S6K = ribosomal p70S6 kinase.

The cell replication process is controlled by mtor through two downstream pathways (Figure 3) mediated by two key proteins: 4EBP1 (translation initiation factor 4E binding protein 1) and p70S6K1 (ribosomal p70S6 kinase). When 4EBP1 is activated by mtor, it dissociates from eIF-4E (eukaryotic translation factor), and leads to cap-dependent messenger (mrna) translation. These mrna encode for c-Myc, cyclin D1, ornithine decarboxylase, and hypoxia-inducible factor (hif), leading to upregulation of a number of growth factors, including the key angiogenic vascular endothelial growth factor (vegf), pdgf, and transforming growth factor (tgf) 11. Synthesis of hif is therefore partly regulated by mtor and normally degraded by the *VHL* (von Hippel–Lindau) gene and its proteins. Dysregulation of the *VHL* gene, seen in clear-cell renal cell carcinomas, results in hifα overexpression and in increased vegf, pdgf, and tgf. Overexpression of hif can therefore be controlled by mtor inhibition. Activation of the p70S6K1 pathway leads to the translation of mrna that encodes ribosomal proteins, elongation factors, and other proteins that are necessary for movement from the G1 phase to the S phase of the cell cycle.

Substantial preclinical and clinical data have shown the pten/pi3k/Akt/mtor pathway to be a major oncogenic pathway in the development of some of the most common cancers and a major therapeutic target in the treatment of human malignancy.

## 3. INHIBITORS OF mTOR

### 3.1 Sirolimus

Sirolimus (Rapamune: Wyeth, Madison, NJ, U.S.A.), initially discovered as an antifungal antibiotic from soil on Rapa Nui (formerly called Easter Island), was recognized in its early development to have anticancer activity (at high doses in murine models 12) and a significant immunosuppressive effect, which although detrimental in fighting infections, proved to be beneficial in transplantation 13. After its approval for transplant immunosuppression in 1999, sirolimus was found to have a significant antitumour effect in experimental models of rcc. *In vitro*, sirolimus changed murine renal cancer cells from an invasive to a noninvasive phenotype, reducing spread and metastatic progression. It also reduced cyclin D1 and increased p27^Kip1^, inhibiting G1-to-S transition 14 and potentially slowing tumour growth 15.

In several murine models of rcc tumour progression, sirolimus was shown to slow tumour growth and metastatic progression and to prolong survival, even in the presence of pro-oncogenic cyclosporine 14. Further studies examining human rcc pulmonary metastases in mice with severe combined immunodeficiency demonstrated the antitumour efficacy of sirolimus with significant reduction of the pro-oncogenic cytokines tgfβ and vegf 16. In contrast to cyclosporine (which has been shown to promote tgfβ), sirolimus has been shown to have an anti-angiogenic effect through downregulation of vegf 15 and potentially a specific effect on tumour vessel thrombosis, which may also contribute to its anti-neoplastic action through an indirect mechanism 17. Blockade of mtor has an inhibitory effect on activity by upstream molecules such as Akt, which occur in some cancers (for example, Kaposi sarcoma) because of loss of regulation by the tumor suppressor pten 9

### 3.2 Everolimus

Everolimus (RAD001: Novartis Pharmaceuticals, St. Louis, MO, U.S.A.) an oral mtor inhibitor, has been shown to have both immunosuppressant and anti-neoplastic properties. At the 2006 annual meeting of the American Society for Clinical Oncology (asco), Amato and colleagues presented a phase ii trial in patients with rcc treated with everolimus after cytokine or cytotoxic therapy. In that study, 25 patients receiving a daily dose of 10mg showed a median progression-free survival (pfs) of more than 9 months 18. In phase i and ii trials, the most common adverse events with everolimus were rash (46%), stomatitis or mucositis (40%), fatigue (32%), and nausea (25%). The most common laboratory abnormalities were anemia (9%), hypercholesterolemia (9%), hyperglycemia (8%), thrombocytopenia (7%), hypertriglyceridemia (3%), and leucopenia or neutropenia (2%).

These phase i and phase ii data in patients with advanced rcc identified significant activity that led to a subsequent larger phase iii second-line trial. In that trial 19, everolimus delayed disease progression in mrcc patients who had progressed on previous targeted therapy (sorafenib, sunitinib). The 410 study patients were randomized 2:1 (everolimus:placebo). The primary endpoint was pfs, with 290 events required to achieve 90% power. Secondary endpoints were safety, response, patient-reported outcomes, and os. Key eligibility criteria were mrcc with clear-cell component, measurable disease, and progressive disease at or within 6months of treatment with sunitinib, sorafenib, or both. (Prior bevacizumab and cytokine treatment were also permitted.) Crossover was allowed in the study, meaning that patients who progressed on therapy were unblinded and allowed to receive everolimus if they had been randomized to placebo.

Of the 410 patients randomized, 272 received everolimus 10mg daily, and 138 received placebo. Median age was 61 years in the treatment group and 60 years in the placebo group. main sites of metastasis were lung (73%, 81%), bone (37%, 31%), and liver (35%, 36%). more than 90% of the patients in both arms had more than one site of metastatic disease. Patients with prior nephrectomy constituted 96% of the everolimus arm and 95% of the placebo arm, and were otherwise comparable. In the everolimus arm, 46% of patients had been treated with sunitinib, 28% with sorafenib, and 28% with both agents. Patient discontinuation in the everolimus arm was 31% because of progressive disease, 10% because of adverse events, and 3% because of death. In the placebo arm, 73% patients discontinued because of progressive disease, 1% because of adverse events, and 2% because of death.

Interim analysis showed that median pfs was 4 months in the everolimus arm and 1.9 months in the control arm, a statistically significant difference (*p* < 0.001). Because of crossover, 80% patients on placebo switched to everolimus, and so os did not show a significant difference.

Inhibition of mtor with everolimus was fairly well tolerated. Common side effects were mouth ulcers or stomatitis (40% vs. 8% placebo), asthenia or fatigue (28% vs. 24%), rash (25% vs. 4%), diarrhea (17% vs. 3%), anorexia (16% vs. 6%), nausea (15% vs. 8%), vomiting (12% vs. 4%), cough (12% vs. 4%), peripheral edema (10% vs. 3%), pneumonitis (8% vs. 0%), and dyspnea (8% vs. 2%). Main laboratory abnormalities (all grades) included anemia (91% vs. 76%), lymphopenia (42% vs. 29%), thrombocytopenia (20% vs. 2%), and neutropenia (11% vs. 3%). Hypercholesterolemia, hypertriglyceridemia, and hyperglycemia were higher in the everolimus arm than in the placebo arm, as was expected, given the mtor-inhibitor class of the drug.

Based on these data showing statistically significant improvement in pfs as compared with placebo, everolimus established clinical benefit as a second-line therapy in patients who progress on first-line targeted therapy, including sunitinib and sorafenib. Everolimus can be proposed as the new standard of care in the second-line setting for patients progressing on targeted therapy with vegf inhibitor.

### 3.3 Temsirolimus

Temsirolimus, a soluble ester analog of rapamycin, was selected for development as an anticancer agent based on its prominent antitumour profile and favourable pharmacologic and toxicologic characteristics in preclinical studies 20. Compared with rapamycin, temsirolimus was found to have improved aqueous solubility, bioavailability, and stability as an anticancer agent 21.

Temsirolimus is a specific inhibitor of mtor kinase 22 that binds to an intracellular protein, Fkbp12, which in turn forms a complex that inhibits the mtor pathway 23. By inhibiting mtor signalling, temsirolimus inhibits the translation of mrna that encodes proteins required for G1 progression and S-phase initiation, and that mediate cell growth and angiogenesis 24. In phase i studies, antitumour activity in a number of cancer types was observed over a broad dose range (Table i).

**FIGURE 4:**
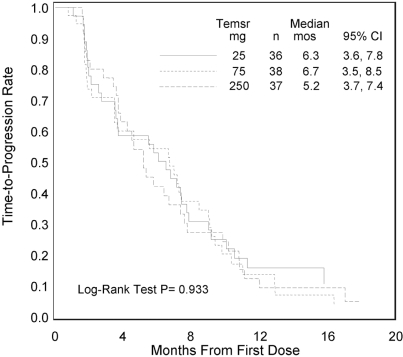
Median time to tumour progression in patients randomly assigned to receive 25 mg, 75 mg, or 250 mg of CCI-779 (temsirolimus) weekly 29.

Although mutations in *PTEN* have not been detected, *PTEN* gene expression is often downregulated in rcc 29. A phase ii single-agent study was conducted to evaluate the efficacy, safety, and pharmacokinetics of CCI-779 in patients with advanced refractory rcc. This randomized double-blind multicentre trial in 111 patients with cytokine-refractory mrcc included patients with poor-risk features. Patients were randomly assigned to receive 25 mg, 75 mg, or 250 mg CCI-779 weekly as a 30-minute intravenous (IV) infusion. Patients were evaluated for tumour response, time to tumour progression (ttp), survival, and adverse events. The CCI-779 produced an objective response rate of 7% (1 complete response and 7 partial responses) and minor responses in 26% of the study patients. Median ttp was 5.8 months, and median survival was 15.0 months (Figure 4). Within each risk group, the median survivals of patients at each dose level were similar.

The most frequently occurring adverse events of all grades related to CCI-779 were maculopapular rash (76%), mucositis (70%), asthenia (50%), and nausea (43%). Reasons for dose reductions included thrombocytopenia (20%), mucositis (16%), hypertriglyceridemia (5%), and neutropenia (1%). In 19% of patients, treatment was discontinued because of drug-related adverse events. Maculopapular rash (5 patients) was the most frequent reason for treatment discontinuation. Pneumonitis was seen in 6 patients, with 2 being withdrawn from study, 2 worsening after drug restart, and 2 having no recurrence of pneumonitis after drug restart. Retrospectively, 49 patients were categorized as being poor-prognosis patients. The CCI-779–treated patients in this poor-risk group experienced a median os that was longer by a factor of 1.7 than that experienced by the first-line interferon-treated poor-risk group reported by Motzer *et al.* 30 (Table ii).

In a phase i study, the maximal tolerated dose of a combination of temsirolimus with interferon in advanced rcc patients was temsirolimus 15 mg IV once weekly with interferon 6×10^6^ IU subcutaneously 3 times weekly 31. The significant activity noted in patients with poor-prognosis features led to a major phase iii trial in advanced rcc patients with poor prognostic features. Patients with advanced rcc and no prior systemic therapy were enrolled in the open-label study if they had at least 3 of the following 6 Mekhail poor-prognosis risk factors:

- Lactate dehydrogenase level greater than 1.5 times the upper limit of normal
- Hemoglobin below the lower limit of normal
- Corrected calcium greater than 10 mg/dL
- Time from diagnosis to first treatment under 1 year
- Karnofsky performance status 60–70
- Disease metastatic to multiple organs

At 209 sites in 26 countries, 626 patients were enrolled, of whom 67% had undergone prior nephrectomy. That proportion of patients with nephrectomy was much lower than the more than 90% of patients with prior nephrectomy in the sunitinib and sorafenib registration trials, likely because the temsirolimus–interferon patients had a poor prognosis and may not have been able to tolerate surgical intervention.

Patients were randomized (1:1:1) to one of 3 arms:

- interferon up to 18×10^6^ IU subcutaneously 3 times weekly (arm 1),
- temsirolimus 25 mg IV once weekly (arm 2), or
- temsirolimus 15 mg IV once weekly with interferon 6×10^6^ IU subcutaneously 3 times weekly (arm 3).

The primary study endpoint was os, and the study was powered to compare the temsirolimus arms with the interferon-only arm.

The three most frequently occurring adverse events were asthenia (27% in arm 1, 12% in arm 2, 30% in arm 3), anemia (24%, 21%, 39%), and dyspnea (8%, 9%, 11%). Less-common adverse events included nausea (5%, 4%, 2%), vomiting (0%, 1%, 5%), hyperlipidemia (1%, 7%, 2%), hyperglycemia (1%, 10%, 4%), thrombocytopenia (0%, 1%, 9%), and neutropenia (8%, 3%, 14%). The proportion of patients with any grade 3 or 4 adverse event was 85% in arm 1, 69% in arm 2, and 87% in arm 2, with the difference between temsirolimus and interferon and between temsirolimus and temsirolimus–interferon being statistically significant (*p* ≤ 0.001). More patients in the interferon groups discontinued treatment because of adverse events (14, 7, and 22 patients respectively).

**FIGURE 5:**
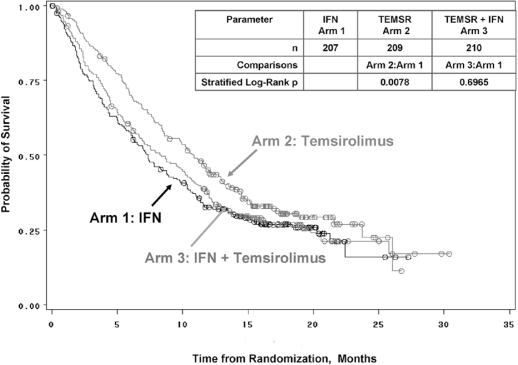
Plot of overall survival, the primary study endpoint in a comparison of temsirolimus and interferon as single agents and in combination 3.

As compared with interferon alfa (*n* = 207), single-agent temsirolimus (*n* = 209) was observed to significantly increase os (10.9 months vs. 7.3 months, *p* = 0.0069) in patients with mrcc judged to be poor-risk. By treatment arm, os was 7.3 months (interferon), 10.9 months (temsirolimus), and 8.4 months (temsirolimus–interferon). Median pfs (Figure 5) was 1.9 months, 3.7 months, and 3.7 months, and objective response (complete and partial responses together) was 7%, 9%, and 11% respectively. The conclusion from the study was that, compared with interferon, single-agent temsirolimus (25 mg IV weekly) significantly increases os, with an acceptable safety profile, in first-line poor-risk advanced rcc patients 3. This study was the first to show a statistically significant benefit in os as well as in pfs with targeted therapy in mrcc patients with poor-prognosis features. It led to approvals by Health Canada and the U.S. Food and Drug Administration of temsirolimus for the first-line treatment of poor-prognosis patients with advanced rcc.

## 4. CONCLUSIONS

The importance of mtor as a novel target in the treatment of advanced rcc has been demonstrated in phase iii clinical trials, both for first-line and second-line treatment after failure of vegfr-rtk inhibitors. The mtor inhibitors temsirolimus and everolimus are well tolerated by patients with advanced rcc. The mtor inhibitors continue to be investigated as single agents and in combination with other anti-neoplastic agents such as rtk inhibitors and vegf inhibitors in rcc and other malignancies.

## Figures and Tables

**TABLE I:** Phase i clinical trials with temsirolimus 5

*Reference*	*Tumour type*	*Pts* *(*n*)*	*Daily dose* *(mg)*	*Regimen*	*Daily* *mtd (mg)*	*Toxicity*	*Activity*
Hidalgo *et al.,* 2000^25^	Renal cell carcinoma, nsclc, soft-tissue sarcomas, cervical and uterine carcinomas	51	0.75–19.1/m^2^	Intravenous days 1–5 every 2 weeks	—a	Hypocalcemia, skin rash, stomatitis, thrombocytopenia	pr: 2% mr or sd: 15%
Farouzesh *et al.,* 2002^26^	Renal cell carcinoma, nsclc, myxoid chondrosarcoma, mesothelioma, leyomiosarcoma	24	25–75	Oral, days 1–5 every 2 weeks	100	Stomatitis, rash, hypertransaminemia	sd: 33%
Punt *et al.,* 2003^27^	Colorectal, gastric carcinoma, esophageal, head-and-neck cancer	28	15–75/m^2^	Temsirolimus in combination with 5-fu/lv	—	Stomatitis/mucositis	pr : 11%
Raymond *et al.,* 2004^21^	Renal cell carcinoma, soft-tissue sarcoma, mesothelioma, nsclc, breast, head-and-neck, melanoma, pancreatic, prostate, neuroendocrine, adrenocortical carcinoma	24	7.5–220/m^2^	Intravenous weekly	—	Skin rash, stomatitis, thrombocytopenia, bipolar disorder	pr : 8.3% mr: 8%
Chang *et al.,* 2004^28^	Glioblastoma	12	250–300	Temsirolimus in combination with enzyme-inducing anti-epileptic drugs, weekly	250	Stomatitis, hypertriglyceridemia	

^a^ 15 mg/m^2^ in heavily pretreated patients.

Pts = patients; mtd = maximal tolerated dose; nsclc = non-small-cell lung cancer; pr = partial response; mr = minor response; sd = stable disease; 5-fu = 5-fluorouracil; lv =leucovorin.

**TABLE II:** Median survival of renal cell carcinoma patients treated with temsirolimus, by risk group 29

*Dose level (mg)*	*Pts (*n*)*	*Good*				*Intermediate*				*Poor*			
		*Pts*		*Survival (months)*		*Pts*		*Survival (months)*		*Pts*		*Survival (months)*	
		*(*n*)*	*(%)*	*Median*	*95%* *ci*	*(*n*)*	*(%)*	*Median*	*95% Ci*	*(*n*)*	*(%)*	*Median*	*95%* *ci*
25	36	2	6	18.4	18.4 to 23.6	14	39	23.0	12.5 to na	20	56	7.1	4.1 to 15.0
75	35^a^	2	6	na	24.1 to na	14	40	20.9	10.4 to 26.1	19	54	8.6	7.3 to 10.3
250	34^a^	4	12	22.4	14.4 to na	20	59	19.3	16.5 to 29.5	10	29	8.4	3.9 to 17.3
All	105	8	8	23.8	17.7 to 27.1	48	46	22.5	16.9 to 25.7	49	47	8.2	7.0 to 10.1

^a^ For 3 patients, data for 1 or more of the prognostic factors were missing. These patients could not be assigned to a risk group.

Pts = patients; ci = confidence interval; na = not available.
